# Putative Role of Fatty Acid Metabolic Therapy Using Ketogenic Diet and HIF-1α Inhibition in Hepatocellular Carcinoma: Evidence from an In Vitro Study

**DOI:** 10.3390/ijms262411769

**Published:** 2025-12-05

**Authors:** Naoya Kimura, Yoshihiko Kitajima, Kazuki Higure, Shohei Matsufuji, Shota Ikeda, Shunsuke Furukawa, Kumpei Yukimoto, Tomokazu Tanaka, Hirokazu Noshiro

**Affiliations:** 1 Department of Surgery, Faculty of Medicine, Saga University, Saga 849-8501, Japan; 22624003@edu.cc.saga-u.ac.jp (N.K.); 22624009@edu.cc.saga-u.ac.jp (K.H.); sm6888@cc.saga-u.ac.jp (S.M.); 23624001@edu.cc.saga-u.ac.jp (S.I.); 25624015@edu.cc.saga-u.ac.jp (S.F.); 25624019@edu.cc.saga-u.ac.jp (K.Y.); f8642@cc.saga-u.ac.jp (T.T.); noshiro@cc.saga-u.ac.jp (H.N.); 2 Department of Laboratory and Research, National Hospital Organization Saga National Hospital, Saga 849-8577, Japan

**Keywords:** hepatocellular carcinoma, ketogenic diet, hypoxia, hypoxia-inducible factor-1, fatty acid oxidation, YC-1, reactive oxygen species

## Abstract

Hypoxia-inducible factor-1 (HIF-1) enhances cancer cell survival in hypoxic conditions. The ketogenic diet (KGD), characterized by low-carbohydrate and high-fat intake, has been widely used for epilepsy treatment and reported to have antitumor potential. However, its impact on hypoxic cancer cells remains poorly understood. This study examined the effects of combining the HIF-1α inhibitor YC-1 with the KD formula KetoCal^®^ on hypoxic hepatocellular carcinoma (HCC) cells. In vitro, HIF-1α knockdown (KD) and scramble control (SC) Hep3B and HepG2 cells were treated with palmitic acid (PA) and/or β-hydroxybutyrate (BOH) to mimic the KGD environment. PA significantly induced cell death and reactive oxygen species (ROS) in hypoxic KD cells, and this effect was further enhanced by BOH. Gene expression analysis indicated that HIF-1 suppresses fatty acid oxidation (FAO) and ketolysis under hypoxia. In vivo, Hep3B cells were implanted into mice fed KetoCal^®^ with or without YC-1. KetoCal^®^ elevated serum BOH and free fatty acids (FFAs), suppressed tumor growth, and increased intra-tumoral acetyl-CoA, ROS, and apoptosis in YC-1-treated tumors. These findings suggest that YC-1 combined with KetoCal^®^ reactivates FAO and ketolysis, promoting acetyl-CoA accumulation and lethal ROS production in hypoxic HCC. This strategy may offer a novel preclinical model for targeting hypoxic tumors.

## 1. Introduction

Primary liver cancer ranks sixth in global cancer incidence and stands as the fourth most common cause of cancer-related death worldwide [1]. It is well-known that hepatocellular carcinoma (HCC) is the most predominant histological subtype, accounting for up to 75% of all primary liver cancer cases [2]. According to the stage or liver function, multidisciplinary treatments are commonly performed in patients with HCC and are entirely managed by highly specialized physicians or surgeons [3]. Currently, several etiologies are being clarified in relation to HCC, including viral infection, alcohol abuse, and fatty liver, but therapeutic strategies have been planned regardless of the etiological causes of HCC [4,5]. Modulations in liver fat metabolism are associated with the development of NASH and NASH-related HCC [6,7].

Clinically, the ketogenic diet—characterized by high fat, very low carbohydrates, and sufficient protein—has long been applied in the management of refractory epilepsy [8,9]. A key mechanism of the ketogenic diet involves replacing glucose with ketone bodies—such as β-hydroxybutyrate (BOH) and acetoacetate (AcAc)—as the primary fuel source for energy metabolism [10]. The ketogenic diet has emerged as a promising metabolic intervention in oncology, aiming to disrupt the altered energy metabolism of cancer cells. Cancer cells primarily rely on aerobic glycolysis rather than oxidative phosphorylation (OXPHOS) for energy production even under normoxia, which is known as the Warburg effect [11]. By limiting glucose availability, the ketogenic diet restricts energy supply, thereby inhibiting tumor growth [12]. Additionally, emerging studies have reported that the ketogenic diet exerts antitumor effects through alternative mechanisms, including the reduction in oxidative stress through the antioxidant effects of ketone bodies [13] and the inhibition of the mTOR signaling pathway [14]. However, the anticancer mechanism underlying the ketogenic diet remains unclear [15,16,17].

The progression of solid tumors, including HCC, is strongly influenced by hypoxic conditions [18]. Hypoxia-inducible factor-1α (HIF-1α) is upregulated under hypoxic conditions [18]. HIF-1α forms a heterodimer with an oxygen-insensitive subunit (HIF-1β) [19]. HIF-1(HIF-1α/HIF-1β heterodimer complex) plays a significant role in metabolic reprogramming and cellular proliferation under hypoxic conditions [20,21]. Under hypoxic conditions, HIF-1 reprograms glucose metabolism by enhancing the expression of key glycolytic genes, thereby shifting energy production from mitochondrial respiration to anaerobic glycolysis [18,20,21]. Mitochondrial respiration under hypoxia also produces reactive oxygen species (ROS) [18]. The electron transport chain (ETC) within mitochondria produces most of the intracellular ROS as part of the OXPHOS process [18]. A previous study demonstrated that the suppression of mitochondrial complex I activity within the ETC plays a pivotal role in ROS generation under acute hypoxic conditions in endothelial cells [22]. Mitochondrial overproduction of ROS may cause oxidative damage to DNA, proteins, and lipids, ultimately triggering cell death [20,21]. Hence, in hypoxic tumors, the HIF-1-mediated enhancement of anaerobic glycolysis serves as a cellular adaptation to minimize mitochondrial ROS accumulation [18,20,21]. Moreover, recent studies reported that HIF-1 upregulates the expression of genes that promote fatty acid (FA) synthesis and downregulates FAO-related genes, in favor of prioritizing glucose metabolism in hypoxic cancer cells [23]. Focusing on the essential role of HIF-1 in suppressing FAO under hypoxia, we recently demonstrated that the combination of palmitic acid (PA) and HIF-1 inhibition suppressed the proliferation of HCC cells. That study showed that FAO is suppressed under hypoxic conditions; however, the inhibition of HIF-1 alleviates this suppression, resulting in the restoration of FAO activity. Consequently, ROS production increased, leading to the induction of apoptosis [24]. Therefore, we hypothesized that the overload of ketone bodies as well as FA by a ketogenic diet combined with HIF-1 inhibitor treatment might exert antitumor effects in HCC, which survives and proliferates in hypoxic environments. To date, no studies have evaluated the antitumor effects of the combination of a ketogenic diet and inhibition of HIF-1, a key factor in cancer metabolism under hypoxic environments. In this study, we explored the mechanism underlying the antitumor effects of this combination treatment on HCC cells using in vitro and in vivo analyses.

## 2. Results

### 2.1. Effect of HIF-1α Knockdown (KD) on ROS Production and Cell Growth in a HCC Cell Line (Hep3B and HepG2) with Treatment of FA and/or Ketone Bodies Under Hypoxic Conditions

**Figure 1:**
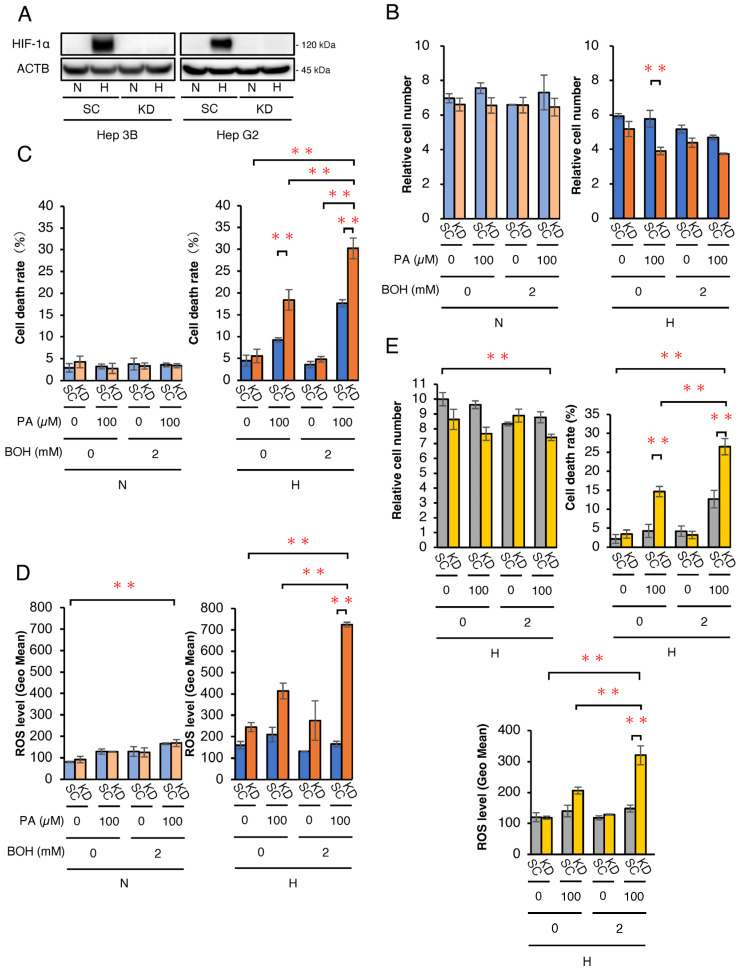
Biological effects of PA and/or BOH treatment in HIF-1-deficient Hep3B and HepG2 cells under normoxic and hypoxic conditions. (**A**) Protein expression analysis under hypoxic conditions by Western blotting in scramble control (SC) and HIF-1α knockdown (KD) cells. (**B**,**C**) For the assessment of cell growth and viability, 5.0 × 10^4^ of SC and HIF-1α KD-Hep3B cells were treated with PA at 0, 100 μM and/or BOH at 0, 2 mM under normoxic (N) and hypoxic (H) conditions for 48 h. (**B**) The mean numbers of living cells are plotted on the graph. (**C**) The cell death rate was estimated by the number of dead cells/total cells and plotted. (**D**) Assessment of ROS levels in Hep3B-SC and -KD cells with treatment by PA at 0, 100 μM and/or BOH at 0, 2 mM for 48 h. (**E**) Relative cell number, cell death rate, and ROS levels under hypoxic conditions in HepG2-SC and -KD cells treated with PA (0 or 100 μM) and/or BOH (0 or 2 mM) for 48 h. Data are presented as mean ± SEM (*n* = 3 independent experiments). Statistical significance was determined using one-way ANOVA followed by Tukey’s post hoc test. N.S., not significant; ** *p* < 0.01.

We first investigated the in vitro effects of ketone bodies and/or FA on Hep3B- and HepG2-HIF-1α KD (KD) cells, in which HIF-1α expression was abrogated by shRNA under hypoxic conditions (Figure 1). A Western blot analysis confirmed that the HIF-1α protein expression was effectively knocked down in KD Hep3B and HepG2 cells under hypoxia (1% O2) (Figure 1A). To simulate a ketogenic diet, 100 μM palmitic acid (PA) and/or 2 mM β-hydroxybutyrate (BOH) were added to the culture medium. KD and control scramble (SC) cells were treated with PA, BOH, PA + BOH, or untreated (control) under normoxia (20% O_2_) and hypoxia (1% O_2_) for 48 h. A cell growth analysis showed no difference between the SC and KD of Hep3B cells under normoxic conditions (Figure 1B). However, under hypoxic conditions, cell numbers were significantly lower in KD cells than in SC cells treated with PA. The cell viability analysis showed no differences under normoxic conditions (Figure 1C). Notably, under hypoxic conditions, PA-treated KD cells exhibited a significantly higher cell death rate than SC cells, which was further increased by PA + BOH treatment. BOH alone did not affect cell death in hypoxic KD cells. While PA and PA + BOH increased cell death in hypoxic SC cells, the effect was more pronounced in KD cells. These results suggest that PA strongly induces cell death in hypoxic Hep3B-KD cells, and BOH further enhances this effect. An ROS analysis revealed no differences between SC and KD cells under normoxic conditions (Figure 1D). Under hypoxic conditions, PA- and PA+BOH-treated KD cells produced significantly higher ROS than SC cells, with BOH exhibiting an additive effect on PA-induced ROS production. In HepG2 cells, a significant increase in both cell death rate and ROS levels was also observed in KD cells under hypoxic conditions following the addition of PA and PA+BOH (Figure 1E).

**Figure 2:**
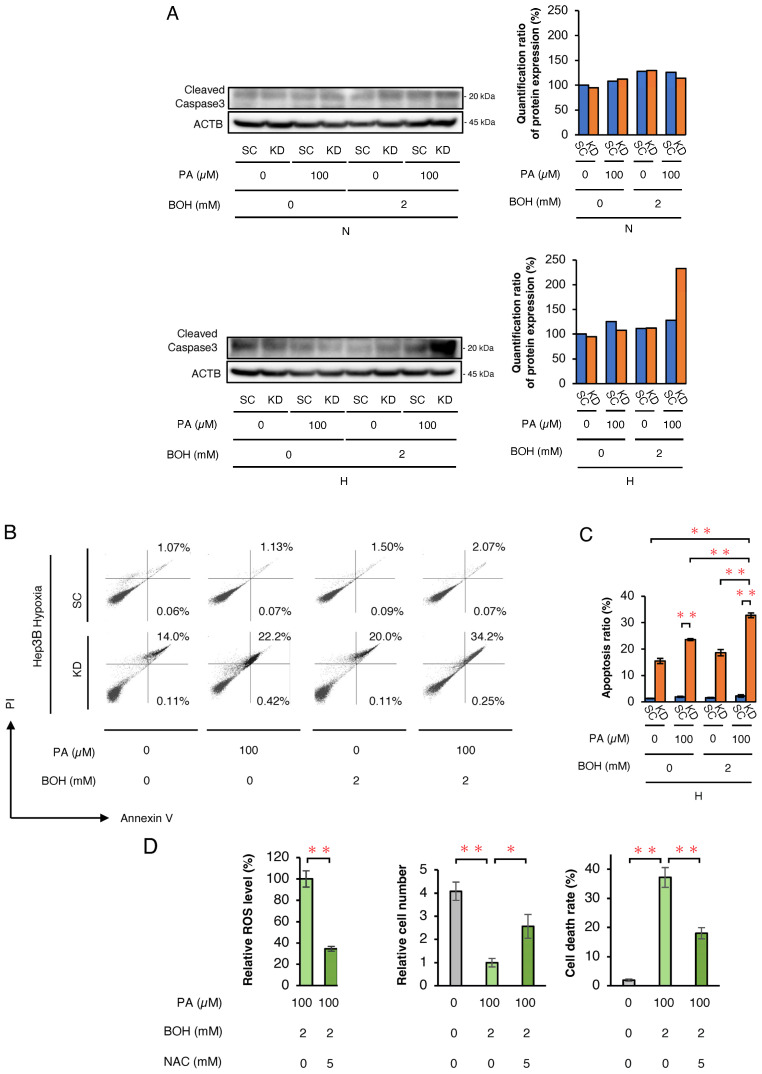
The analysis of cell apoptosis in Hep3B-SC and -KD cells and evaluation of the inhibitory effects of an ROS scavenger on cell proliferation, cell death rate, and ROS production. (**A**) The Western blot analysis of an apoptosis marker (cleaved caspase-3) in SC and KD cells, treated with PA and/or BOH at the indicated concentrations under normoxic and hypoxic conditions for 48 h. Quantified value of cleaved caspase-3 expression normalized to ACTB is plotted on graph and shown to right of the blots. (**B**) The flow cytometric analysis of apoptosis using Annexin V and PI in SC and KD cells treated with PA and/or BOH at the indicated concentrations under hypoxic conditions for 48 h. Proportions of apoptotic cells are indicated by percentage of Annexin V+/PI− or Annexin V+/PI+. (**C**) The results shown in Figure 2B are quantified and plotted in Figure 2C (*n* = 5). (**D**) The analysis of changes in ROS production, cell number, and cell death rate under treatment with ROS scavenger NAC (5 mM). Hep3B-KD cells treated with PA (100 μM) and BOH (2 mM) were incubated under hypoxic conditions for 48 h with or without NAC (5 mM). Data are presented as mean ± SEM (*n* = 3 independent experiments). Statistical significance was determined using one-way ANOVA followed by Tukey’s post hoc test. * *p* < 0.05, ** *p* < 0.01.

Next, Western blotting for cleaved caspase-3 was performed to assess cell apoptosis (Figure 2A). Under normoxic conditions, no differences were observed between Hep3B- SC and -KD cells. However, under hypoxic conditions, cleaved caspase-3 expression was markedly higher in KD cells treated with PA + BOH. Furthermore, to more precisely assess apoptosis induced by PA and/or BOH treatment, we conducted a flow cytometric analysis of apoptosis using Annexin V and PI staining in Hep3B-KD and -SC cells under hypoxic conditions. The proportion of apoptotic cells significantly increased with the addition of PA and was further elevated by combined treatment with PA and BOH (Figure 2B,C). These findings indicate that under hypoxic conditions, HIF-1α abrogation in KD cells may lead to excessive ROS production with PA and PA + BOH, subsequently inducing cell death by apoptosis. To confirm ROS-induced apoptosis with PA+BOH treatment, we treated hypoxic Hep3B-KD cells with the ROS scavenger N-acetylcysteine (NAC). Flow cytometric analysis demonstrated that intracellular ROS levels were effectively decreased by NAC in PA+BOH-treated KD cells under hypoxic conditions (Figure 2D). NAC treatment also reversed the PA+BOH-induced decrease in the viable cell number and increase in the cell death rate. These results clearly showed that PA+BOH-induced apoptosis in hypoxic KD cells was caused by excessive ROS production.

### 2.2. Analysis of the Expression of Genes Related to Ketone Body and Fatty Acid Metabolism

**Figure 3:**
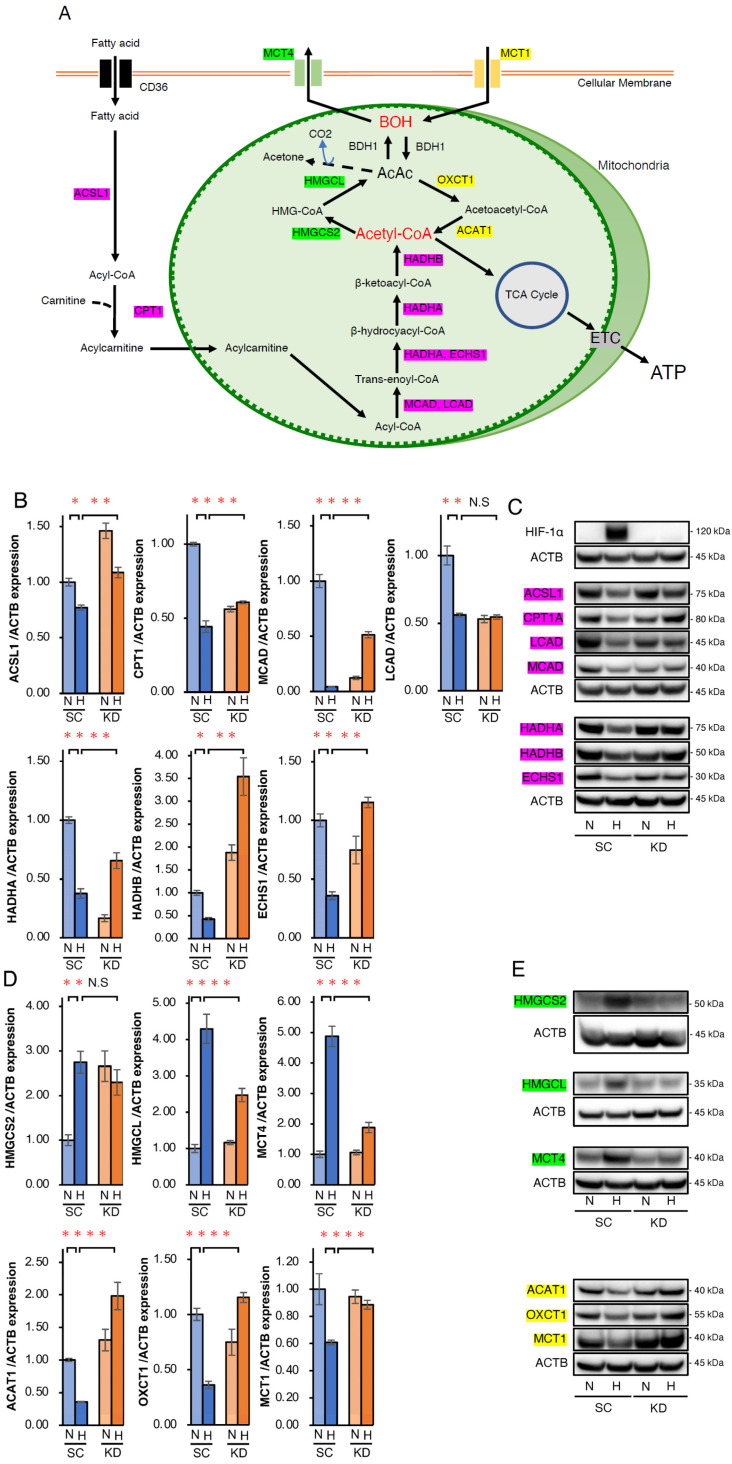
Analysis of the expression of key enzymes related to fatty acid oxidation (FAO) and ketone body metabolism in SC and KD cells under normoxic and hypoxic conditions. (**A**) Schematic diagram of the metabolic pathways related to FAO and ketone body metabolism. Key enzymes of FAO (red box), ketogenesis (green box), ketolysis (yellow box), and the corresponding intermediates are shown. TCA, tricarboxylic acid; ETC, electron transport chain. (**B**) qRT-PCR of key enzyme genes related to fatty acid β-oxidation (FAO), including *ACSL1*, *CPT1A*, *MCAD*, *LCAD*, *HADHA*, *HADHB*, and *ECHS1* in SC and KD cells under normoxic (N) or hypoxic (H) conditions for 24 h. (**C**) Western blot analysis of ACSL1, CPT1A, MCAD, LCAD, HADHA, HADHB, and ECHS1 in SC and KD cells under normoxic (N) or hypoxic (H) conditions for 48 h. (**D**) qRT-PCR of key enzyme genes related to ketogenesis (*HMGCS2*, *HMGCL*, and *MCT4*) and ketolysis (*ACAT1*, *OXCT1*, and *MCT1*) under normoxic conditions (N) or hypoxia (H) for 24 h. (**E**) Western blot analysis of HMGCS2, HMGCL, MCT4, ACAT1, OXCT1, and MCT1 in SC and KD cells. Data are presented as mean ± SEM (*n* = 3 independent experiments). Statistical significance was determined using one-way ANOVA followed by Tukey’s post hoc test. N.S., not significant; * *p* < 0.05, ** *p* < 0.01.

To elucidate how HIF-1α abrogation and PA+BOH treatment induced apoptosis in hypoxic Hep3B cells, we analyzed the expression of genes related to FA and ketone metabolism. Figure 3A illustrates the metabolic pathways of FAO, ketogenesis, and ketolysis, highlighting the key intermediates and enzymes. FAO involves the acyl-CoA synthetase long-chain family member 1 (*ACSL1*)-mediated conversion of FA to acyl-CoA, followed by carnitine palmitoyltransferase (*CPT1A*)-mediated transport into the mitochondria as acylcarnitine. Acyl-CoA is sequentially metabolized to acetyl-CoA via medium-chain acyl-CoA dehydrogenase (*MCAD*), long-chain acyl-CoA dehydrogenase (*LCAD*), hydroxyacyl-CoA dehydrogenase trifunctional multienzyme complex subunit alpha (*HADHA*), enoyl-CoA hydratase, short chain 1 (*ECHS1*), and hydroxyacyl-CoA dehydrogenase trifunctional multienzyme complex subunit beta (*HADHB*) (Figure 3A). Ketogenesis converts acetyl-CoA to BOH via 3-hydroxy-3-methylglutaryl-CoA synthase 2 (*HMGCS2*) and 3-hydroxy-3-methylglutaryl-CoA lyase (*HMGCL*), with beta-hydroxybutyrate dehydrogenase type 1 (*BDH1*) facilitating AcAc-BOH interconversion and BOH exported via monocarboxylate transporter 4 (*MCT4*). Conversely, ketolysis imports BOH via monocarboxylate transporter 1 (*MCT1*), converting it back to AcAc, acetoacetyl-CoA, and acetyl-CoA via *BDH1*, 3-oxoacid CoA-transferase 1 (*OXCT1*), and acetyl-CoA acetyltransferase 1 (*ACAT1*), respectively (Figure 3A). qRT-PCR showed that the mRNA expression of FAO-related genes including the significantly suppressed *ACSL1*, *CPT1*, *MCAD*, *LCAD*, *HADHA*, *HADHB*, and *ECHS1* was significantly suppressed in SC cells under hypoxia compared with normoxia (Figure 3B). However, the expression of these genes was significantly increased under hypoxia in KD cells relative to SC cells, except for *LCAD* (Figure 3B). Western blotting confirmed these trends at the protein level (Figure 3C), and suggested that HIF-1 negatively regulated FAO under hypoxic conditions. For ketone metabolism, qRT-PCR showed the upregulation of ketogenesis promoting *HMGCS2*, *HMGCL*, and *MCT4* in SC under hypoxic conditions, with significantly reduced *HMGCL* and *MCT4* induction in KD cells, except for *HMGC2* (Figure 3D). Conversely, *ACAT1*, *OXCT1*, and *MCT1* expressions, which are involved in ketolysis, were suppressed in SC cells but significantly restored in KD cells under hypoxic conditions (Figure 3D). Similar gene expression findings were further confirmed at the protein level by Western blotting (Figure 3E). Collectively, these data suggested that HIF-1 promotes ketogenesis while suppressing FAO and ketolysis in hypoxic Hep3B cells (Table 1). In addition, the HIF-1-dependent suppression of FAO and ketolysis-related genes in hypoxic HepG2 cells was also suggested in WB analysis, whereas the hypoxic induction of ketogenesis-related *HMGCS2* and *HMGCL* expressions by HIF-1 was not determined (Supplementary Figure S1 and Supplementary Table S1).

### 2.3. Ketogenic Diet Suppressed Growth of KD Tumors in Nude Mice

**Figure 4:**
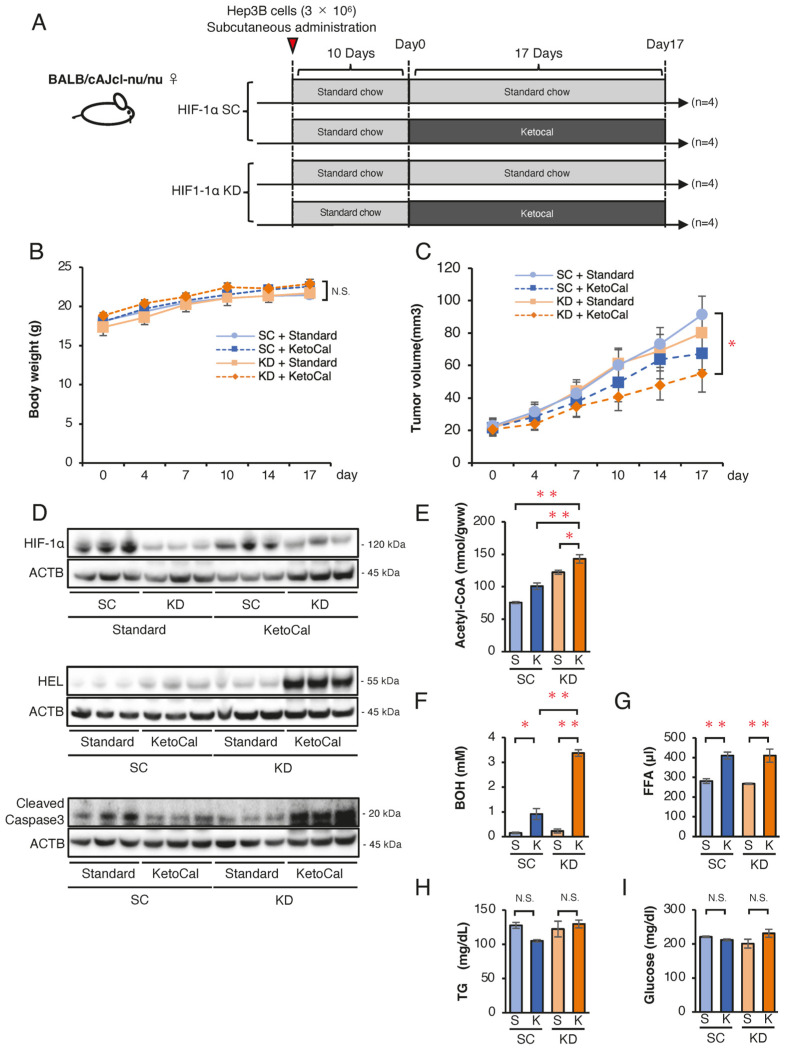
Effects of a ketogenic diet KetoCal^®^ on tumor growth and metabolic markers in vivo. (**A**) Experimental schedule of Hep3B-SC and -KD tumors in nude mice fed standard chow (S) or KetoCal^®^ (K). The four treatment groups (SC tumors with standard chow [*n* = 4], SC tumors with KetoCal^®^ [*n* = 4], KD tumors with standard chow [*n* = 4], and KD tumors with KetoCal^®^ [*n* = 4]) are shown. (**B**) Body weight transition of tumor-bearing mice during the 17-day treatment period. (**C**) Transition of the mean tumor volume of KD and SC tumors (SC + standard chow, SC + ketogenic chow, KD + standard chow, and KD + ketogenic chow). Each group consisted of four mice, with bilaterally subcutaneous implantation, resulting in 8 tumors analyzed per group. (**D**) Western blotting of HIF-1α, HEL, and the cleaved caspase-3 expression in KD and SC xenograft tumors from the four treatment groups. (**E**) Intratumoral acetyl-CoA levels in KD and SC xenograft tumors from the four treatment groups. (**F**–**I**) Blood examination of serum β-Hydroxybutyrate (BOH), free fatty acid (FFA), triglyceride (TG), and glucose levels in mice from the four treatment groups. Tumors were randomly selected from each group and subjected to analyses. The presented Western blot images are cropped from the original full-length blots, which are provided in Supplementary Information. Data are presented as mean ± SEM. One-way ANOVA with Tukey’s post hoc test or unpaired Student’s *t*-test was applied as appropriate. N.S., not significant; * *p* < 0.05, ** *p* < 0.01.

To assess whether a high-fat, very-low-carbohydrate ketogenic diet promotes apoptosis in HIF-1α-deficient tumors in vivo, we conducted a comparative xenograft study. Figure 4A shows nude mice (*n* = 16) subcutaneously inoculated with SC or KD cells and fed a standard chow diet for 10 days. Tumor formation was confirmed on day 10 (designated as day 0), and mice were then divided into four groups (*n* = 4 per group): SC + standard chow, SC + KetoCal^®^, KD + standard chow, and KD + KetoCal^®^, and observed for 17 days. On day 17, body weight remained unchanged across the groups (Figure 4B). Tumor growth did not differ between the standard chow and KetoCal^®^ in SC tumors. However, the tumors in KD mice fed KetoCal^®^ were significantly smaller than those in SC mice fed standard chow (Figure 4C). The expression of HIF-1α was reduced in KD tumors, indicating spontaneous hypoxia within SC tumors, and HIF-1α knockdown was preserved in KD in vivo (Figure 4D). The expression of an ROS marker hexanoyl lysine (HEL) and cleaved caspase-3 was the highest in KD tumors + KetoCal^®^ (Figure 4D). Intratumoral acetyl-CoA levels were elevated in SC and KD tumors treated with KetoCal^®^ relative to standard chow, with KD tumors showing the highest levels (Figure 4E). Serum BOH and FFA levels were significantly higher in KetoCal^®^-fed mice than in standard chow-fed mice, and BOH levels were highest in KD mice (Figure 4F,G). Meanwhile, serum triglyceride (TG) and glucose levels remained unchanged in all groups (Figure 4H,I). Collectively, these results suggest that HIF-1α knockdown combined with KetoCal^®^ suppressed HCC tumor growth by increasing intra-tumoral acetyl-CoA and ROS, and led to apoptosis. These metabolic shifts had no adverse effects on body weight or survival in mice.

### 2.4. Ketogenic Diet Combined with HIF-1 Inhibitor YC-1 Suppresses the Tumor Growth of Hep3B Cells in Nude Mice

**Figure 5:**
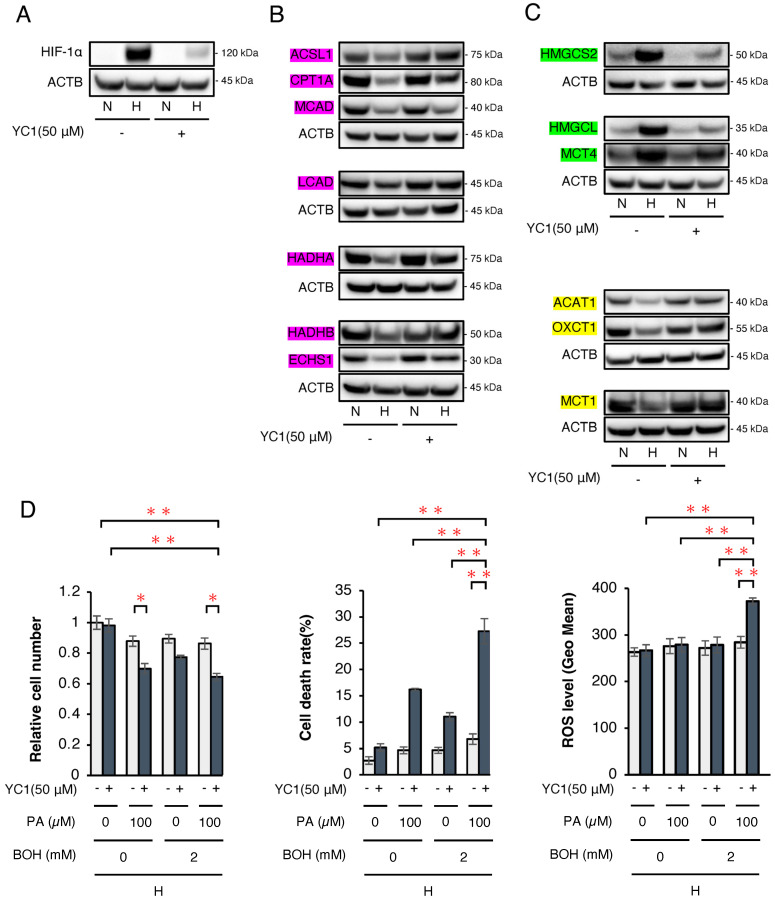
Analysis of the expression of key enzymes related to FA and ketone body metabolism in Hep3B cells with or without treatment by a HIF-1α inhibitor (YC-1). (**A**) Western blotting of the HIF-1α protein expression in wild-type Hep3B cells with/without YC-1 (50 µM) under normoxic (N) or hypoxic (H) conditions for 48 h. (**B**) Western blotting to analyze the protein expression of FAO-related enzymes (ACSL1, CPT1A, MCAD, LCAD, HADHA, ECHS1, and HADHB; shown by red box), (**C**) ketogenesis-related enzymes (HMGCS2, HMGCL, and MCT4; shown by green box), and ketolysis-related enzymes (ACAT1, OXCT1, and MCT1; shown by yellow box) in Hep3B cells with/without YC-1 (50 µM) under normoxic (N) or hypoxic (H) conditions for 48 h. The presented Western blot images are cropped from the original full-length blots, which are provided in Supplementary Information. For clarity, bands detected from the same membrane are grouped together above the loading control (ACTB). (**D**) Relative cell number, cell death rate, and ROS levels under hypoxic conditions in wild-type Hep3B cells with/without YC-1 (50 µM) treated with PA (0 or 100 μM) and/or BOH (0 or 2 mM) for 48 h. Data are presented as mean ± SEM (*n* = 3 independent experiments). Statistical significance was determined using one-way ANOVA followed by Tukey’s post hoc test. * *p* < 0.05, ** *p* < 0.01.

**Figure 6:**
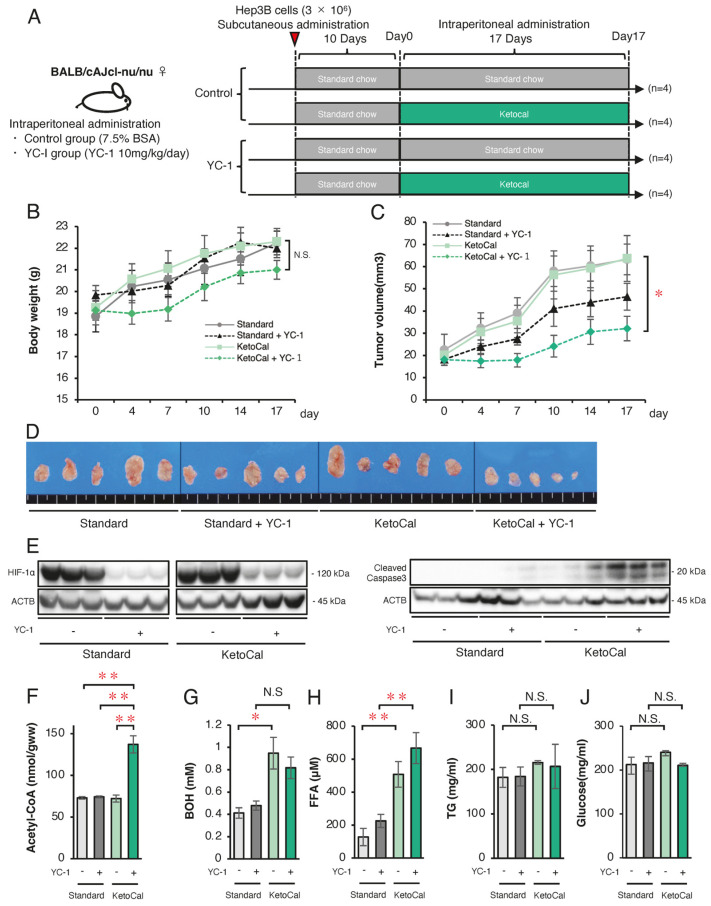
Effect of combined therapy with a ketogenic diet KetoCal^®^ and YC-1 on tumor growth and metabolic markers in vivo. (**A**) Experimental schedule of Hep3B xenograft tumors in the four treatment groups (YC-1[-] with standard chow [*n* = 4], YC-1[+] with standard chow [*n* = 4], YC-1[-] with KetoCal^®^ [*n* = 4], and YC-1[+] with KetoCal^®^ [*n* = 4]). (**B**) Body weight transition of mice during the 17-day treatment period. (**C**) The mean tumor volume of Hep3B xenografts in the four treatment groups. Each group included four mice, with bilaterally subcutaneous implantation, resulting in 8 tumors analyzed per group. (**D**) The macroscopic appearance of tumors excised from mice in the four treatment groups. Each group consisted of four mice with bilateral subcutaneous tumors, resulting in eight tumors per group. Five representative tumors per group are shown in the figure for visual clarity. (**E**) Western blotting of HIF-1α and cleaved-caspase3 protein in Hep3B xenograft tumors excised from mice in the four treatment groups. (**F**) Intra-tumoral acetyl-CoA levels in Hep3B xenograft tumors excised from mice in the four treatment groups. (**G**–**J**) Blood examination of serum β-Hydroxybutyrate (BOH), free fatty acid (FFA), triglyceride (TG), and glucose levels in mice in the four treatment groups. The presented Western blot images are cropped from the original full-length blots, which are provided in Supplementary Information. For clarity, bands detected from the same membrane are grouped together above the loading control (ACTB). Data are presented as mean ± SD (*n* = 3 independent experiments). One-way ANOVA with Tukey’s post hoc test or unpaired Student’s *t*-test was applied as appropriate. N.S., not significant; * *p* < 0.05, ** *p* < 0.01.

Finally, we attempted to conduct a therapeutic model by clarifying whether or not a ketogenic diet combined with a HIF-1α inhibitor 3-(5′-hydroxymethyl-2′-furyl)-1-benzylindazole (YC-1) effectively suppresses HCC tumor growth in mice. First, we analyzed the in vitro protein expression of enzymes related to FA and ketone metabolism in wild-type Hep3B cells under normoxic and hypoxic conditions (Figure 5). Western blotting confirmed that YC-1 strongly inhibited the expression of HIF-1α under hypoxic conditions (Figure 5A). As observed in SC and KD cells, the FAO-related enzyme expression was suppressed under hypoxic conditions, while YC-1 reversed this suppression (Figure 5B). Similarly, the ketogenesis-related enzyme expression increased under hypoxic conditions but was suppressed by YC-1, whereas ketolysis-related enzymes decreased under hypoxic conditions and were restored by YC-1 (Figure 5C). In Hep3B cells, treatment with the HIF-1 inhibitor YC-1 under hypoxic conditions resulted in a significant increase in both cell death rate and ROS levels following the addition of PA or PA+BOH, similar to the effects observed in HIF-1αSC and KD cells (Figure 5D). These findings indicate that YC-1 had comparable effects to those of HIF-1α shRNA in reversing hypoxia-dependent gene regulation. Next, we evaluated tumor suppression by YC-1 and the ketogenic diet in vivo. YC-1 (10 mg/kg/day) was administered intraperitoneally, while the control group received 7.5% BSA. As per the treatment schedule (Figure 6A), Hep3B tumor-bearing mice (*n* = 16) were divided into four groups (*n* = 4 per group): YC-1(−) + standard chow, YC-1(+) + standard chow, YC-1(−) + KetoCal^®^, and YC-1(+) + KetoCal^®^. On day 17, the body weight did not differ among the groups (Figure 6B). However, the tumor volume in YC-1(+) + KetoCal^®^ mice was significantly reduced in comparison to YC-1(−) + standard chow mice (Figure 6C,D). No significant differences were found between the standard chow and KetoCal^®^ in YC-1(-) groups. Although the YC-1(+) + standard chow group showed more of a trend toward smaller tumors than the YC-1(-) + standard chow group, the difference was not statistically significant (Figure 6C,D). In the tumor protein analysis, the expression of HIF-1α was strongly suppressed in YC-1-treated tumors (Figure 6E). The expression of the apoptosis marker cleaved caspase-3 was the highest in YC-1(+) + KetoCal^®^ tumors (Figure 6E). Intratumoral acetyl-CoA levels were also significantly elevated in YC-1(+) + KetoCal^®^ tumors (Figure 6F). Serum BOH and FFA levels were significantly higher in YC-1(-) + KetoCal^®^ and YC-1(+) + KetoCal^®^ groups than in their standard chow counterparts (Figure 6G,H). The serum TG and glucose levels in the KetoCal^®^ and standard chow groups did not differ to a statistically significant extent (Figure 6I,J). Overall, these in vivo findings with YC-1 treatment were mostly consistent with those obtained using HIF-1 KD tumors, supporting the therapeutic potential of combining YC-1 with a ketogenic diet.

## 3. Discussion

To the best of our knowledge, we demonstrated, for the first time, that the antitumor effects resulted from oxidative stress induced by the combination therapy of HIF-1α inhibition and a ketogenic diet in hypoxic HCC cells, which may be a novel therapeutic choice. Cancer cells are characterized by metabolic reprogramming, leading to a high uptake of glucose [25]. This modification, referred to as the Warburg effect, promotes aerobic glycolysis to facilitate ATP production even under normoxic conditions [11]. Under hypoxic conditions, anaerobic glycolysis is activated, leading to further glucose demand, which is essential for survival, by generating ATP through lactate fermentation [26]. From this point of view, previous reports suggested that depleting glucose in cancer cells due to a critical shift in energy metabolism plays a central role in the antitumor effect of a ketogenic diet [27,28,29]. However, emerging studies have reported that alternative mechanisms may also play an essential role in the anticancer effects of a ketogenic diet [13,14]. 

We recently reported that treatment with 100 μM PA induces apoptosis in HIF-1-deficient HCC cell lines under hypoxia, but not normoxia [24]. Other studies have also estimated the BOH concentration in serum to be approximately 1~4 mM in xenograft-bearing mice [30,31,32]. Based on these studies, we first conducted in vitro studies investigating the ability of 100 μM PA and/or 2 mM BOH treatments to induce apoptosis in HIF-1α KD-Hep3B and KD-HepG2 cells. Under hypoxic conditions, the addition of PA to HIF-1α KD cells induced cell death, which was further enhanced by the addition of BOH. ROS production in hypoxic KD cells showed a similar trend. Finally, we confirmed that ROS scavenging by NAC treatment reversed the effect of cell death in Hep3B-KD cells. Hence, we assumed that hypoxia-dependent apoptosis might be induced by excessive ROS production in PA+BOH-treated KD cells.

Next, we conducted a series of expression analyses, which revealed that the mRNA and protein expression of FAO-related enzymes was suppressed in SC cells under hypoxic conditions. Moreover, hypoxia-dependent downregulation of these enzymes was restored in KD cells, suggesting that HIF-1 suppresses FAO in both Hep3B and HepG2 cells under hypoxic conditions by downregulating the expression of FAO-related enzymes. Similarly, we demonstrated that gene expression related to the ketolysis pathway, such as the uptake of ketone bodies and their conversion back to acetyl-CoA, was also downregulated by HIF-1 in HCC cell lines under hypoxia. Conversely, gene expression related to the ketogenesis pathway converting acetyl-CoA to ketone body was upregulated by HIF-1 in hypoxic Hep3B cells. In general, HIF-1 binds to the hypoxic response element (HRE) 5′-A/GCGTG-3′ in the promoter regions of various target genes and transcriptionally upregulates mRNA expression levels [33,34,35,36]. Hence, under hypoxia, HIF-1 may upregulate the mRNA expression of ketogenesis-related enzymes in Hep3B cells by directly binding to the HRE in the promoter region. In contrast, a notable study reported that the transcription factor PGC-1β directly upregulated FAO-promoting *MCAD* and *LCAD* mRNA [37]. Moreover, HIF-1 inhibited the expression of PGC-1β and the upstream factor c-Myc under hypoxic conditions [37]. Based on these findings, the authors suggested that HIF-1-mediated suppression of the axis from c-Myc to PGC-1β is involved in the hypoxic suppression of *MCAD* and *LCAD* [37]. Therefore, HIF-1-dependent suppression of FAO- and ketolysis-related genes, as demonstrated in hypoxic Hep3B and HepG2 cells, may be induced via this mechanism. However, to elucidate the precise mechanism of the HIF-1-regulated gene expression in FA and ketone body metabolism, a promoter analysis, including a CHIP assay, may be necessary for each of the related genes. In addition, beta-hydroxybutyrate dehydrogenase (BDH) is a critical enzyme in ketone body metabolism, facilitating the interconversion between BOH and AcAc [33,34,38]. Several reports have suggested that cancer cells, such as glioma cells, are unable to metabolically utilize ketone bodies because of the absence of *BDH1* [39,40]. In contrast, cancer cells of different origins, including HCC, can take up and metabolize ketone bodies [15,41,42]. Thus, the capability of ketone bodies to be utilized in cancer cells remains controversial. In the present study, we confirmed that *BDH1* was expressed at the mRNA and protein levels in SC and KD from both HCC cell lines, although hypoxia-dependent regulation by HIF-1 was not determined (Supplementary Figure S2). These findings suggest that HCC cells may be capable of taking up ketone bodies from the extracellular environment and producing acetyl-CoA via the ketolytic pathway. Additionally, ketogenesis-related genes were clearly induced by HIF-1α under hypoxia in Hep3B cells; however, this response was not evident in HepG2 cells. This discrepancy may reflect the cell line-specific metabolism of the ketone body.

**Figure 7:**
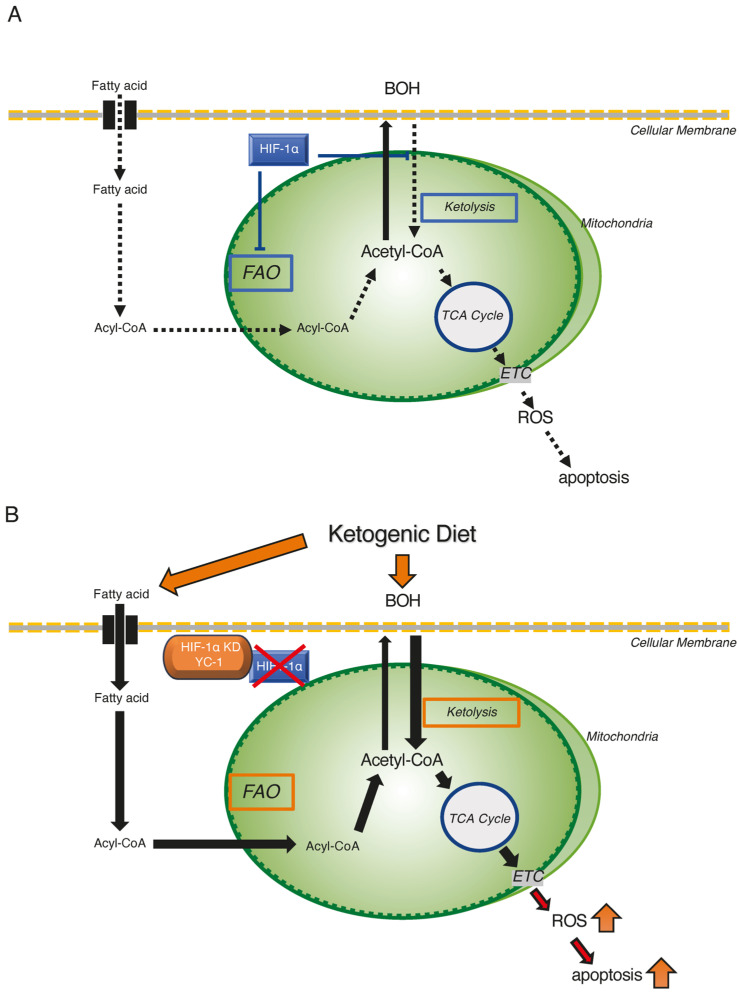
Schematic representation of the ketogenic diet combined with HIF-1α inhibition as a therapeutic strategy targeting hypoxic HCC tumors. (**A**) Metabolic reprogramming of FAO and ketone metabolism by HIF-1α in hypoxic HCC tumor regions. HIF-1α suppresses FAO activity and ketolysis under hypoxic conditions. (**B**) Apoptotic effects of the ketogenic diet combined with HIF-1α inhibition in hypoxic HCC cells. HIF-1α inhibition restores FAO activity and ketolysis by reversing the hypoxia-suppressed expression of critical enzymes. Consequently, the accumulated acetyl-CoA enters TCA cycle, and excess ROS are produced via ETC to induce apoptosis in hypoxic HCC cells. Activated and suppressed metabolisms were shown by red-squared and blue-squared boxes, respectively.

Based on these in vitro findings, we propose a three-step mechanism for the tumor-suppressive effects of combining HIF-1α inhibition with a ketogenic diet. First, we hypothesized that the combination treatment would cause an increase in mitochondrial acetyl-CoA concentrations in hypoxic Hep3B and HepG2 cells, in which both FAO and ketolysis activities were restored. Second, increased mitochondrial acetyl-CoA promotes reactions of the TCA cycle and the ETC. Finally, excessive ROS are produced through the OXPHOS pathway and trigger cellular damage to induce apoptosis (Figure 7).

To further verify these hypotheses, we attempted an in vivo study using a Hep3B xenograft model to assess the tumor-suppressive effect of the combination treatment with HIF-1α inhibition and a ketogenic diet (KetoCal^®^). We designed two animal experiments for HIF-1 inhibition: the HIF-1α KD strain and the HIF-1α inhibitor YC-1. YC-1 is known to strongly inhibit the HIF-1α expression via a unique mechanism of proteasome-independent degradation [43]. Both in vivo experiments demonstrated that the tumor growth was clearly suppressed by the combination of HIF-1α inhibition and KetoCal^®^. The oral intake of KetoCal^®^ significantly increased serum BOH and FFA, compared with the standard chow, in both in vivo models. Furthermore, KetoCal^®^ intake more strongly increased the intra-tumoral acetyl-CoA and cleaved caspase-3 expression in HIF-1α KD and YC-1-treated tumors, compared with other groups.

In mammals, BOH constitutes 70–80% of ketone bodies [44]. A ketogenic diet, low in glucose and high in fat, is absorbed by the intestine and transported to the liver via the portal vein [15]. BOH is generated from acetyl-CoA in hepatocytes due to the limited oxaloacetate availability during the TCA cycle. Owing to the absence of *OXCT1*, normal hepatocytes are unable to convert BOH back to acetyl-CoA, resulting in the circulation of BOH and excess FAs to extrahepatic tissues, including the brain, heart, kidneys, and tumors [15]. In the present study, the oral intake of KetoCal^®^ resulted in significantly higher concentrations of serum BOH and FFA in comparison to normal chow, indicating that BOH is effectively produced in the livers of tumor-bearing mice. Furthermore, the highest concentration of intra-tumoral acetyl-CoA was observed in KetoCal^®^-fed mice, which bore HIF-1α KD tumors or underwent YC-1 treatment. These results suggested a possibility that extracellular BOH and FFA, which originated from the intake of KetoCal^®^, are more highly incorporated into the hypoxic region within the HIF-1α KD or YC-1-treated tumor relative to the SC or YC-1-untreated tumor. Subsequently, lethal ROS are generated in the hypoxic mitochondria of these HIF-1-deficient tumors through the OXPHOS pathway. In addition, serum BOH and FFA levels did not decline in the HIF-1α KD-bearing or YC-1-treated mice with KetoCal^®^ intake, compared with SC-bearing or YC-1-untreated mice. The uptake of BOH and FFA from the serum into Hep3B tumors may be so small that it did not affect their blood concentrations. Meanwhile, a ketogenic diet using KetoCal^®^ did not demonstrate a stronger antitumor effect in SC- or wild-type Hep3B tumor-bearing mice compared to standard chow. Therefore, mono-treatment with the KetoCal^®^ diet may be insufficient to inhibit Hep3B tumor growth within the therapeutic schedule used in the present study.

This study has several limitations that should be acknowledged. First, although we demonstrated alterations in the expression of key enzymes involved in FAO and ketolysis, dynamic assessments of metabolic flux such as FAO and OXPHOS activities (O_2_ consumption rate) by Seahorse device are required. Second, this study did not directly address the assessment of mitochondrial integrity, including mitochondrial membrane potential and electron leak. These functional validations may provide stronger evidence linking acetyl-CoA accumulation to mitochondrial ROS production and apoptosis in HIF-1a-deficient HCC cells under hypoxia.

In conclusion, combination therapy with the HIF-1α inhibitor YC-1 and a ketogenic diet (KetoCal^®^) targets hypoxic regions in HCCs, where FA and ketone bodies are forcedly metabolized into acetyl-CoA through FAO and ketolysis in HIF-1α-depleted cells. Excessive acetyl-CoA enters the TCA cycle and generates lethal ROS via oxidative phosphorylation (OXPHOS). On the other hand, this treatment may not exert harmful effects on normal tissues living under normoxic environments where only a small amount of ROS may be produced. The oral administration of KetoCal^®^ is actually used for the treatment of epilepsy in humans. Thus, the combination treatment with an HIF-1 inhibitor YC-1 + KetoCal^®^ may provide a promising model of an anticancer strategy that targets hypoxic HCC cells. Furthermore, although the current study did not assess the influence of specific etiologies such as viral hepatitis, alcoholic liver disease, or metabolic dysfunction-associated steatotic liver disease, the observed mechanism—targeting hypoxia-driven metabolic vulnerabilities—may potentially be effective regardless of the underlying causes. This suggests that the combination of HIF-1α inhibition and ketogenic dietary intervention could serve as a broadly applicable therapeutic approach for hepatocellular carcinoma.

## 4. Materials and Methods

### 4.1. Cell Lines

The human hepatocellular carcinoma cell lines Hep3B and HepG2 were purchased from the American Type Culture Collection (ATCC, Manassas, VA, USA) and cultured in Dulbecco’s Modified Eagle Medium (DMEM) containing 10% fetal bovine serum (FBS), 100 U/mL penicillin, and 100 µg/mL streptomycin (168–23191, FUJIFILM, Wako, Japan), and incubated at 37 °C in a humidified environment. The cultures were subjected to either normoxic conditions (20% O_2_ and 5% CO_2_) or hypoxic conditions (1% O_2_, 5% CO_2_, and 94% N_2_) as previously described 20.21. HIF-1α KD cells and control SC cells had been established in both Hep3B and HepG2 cell lines as described previously [20,24]. Briefly, transfections were performed using plasmids encoding short hairpin RNA (shRNA) targeting HIF-1α or a scrambled shRNA as a control. Transfected cells were selected with puromycin (1.5 mg/mL in the culture medium) and designated as HIF-1 KD and SC cells, respectively.

### 4.2. Preparation of PA Solution

The preparation of the palmitate/bovine serum albumin (BSA) solution followed the protocol reported in a previous study [45]. Briefly, a 100 mM PA stock solution was prepared by dissolving PA in 0.1 M NaOH in a shaking water bath at 70 °C for 10 min. For in vitro studies, this stock solution was diluted 1:20 with 10% BSA solution at 55 °C in water bath by vortex mixing. The resulting solution was further diluted in DMEM to a final concentration of 100 μM.

### 4.3. HIF-1α Inhibitor

YC-1 (Sigma-Aldrich, St. Louis, MO, USA) was used as the HIF-1α inhibitor. The compound was diluted in DMSO, according to the manufacturer’s protocol, to prepare a 10 mM stock solution. For the in vitro experiments, the stock solution was mixed with DMEM to achieve a final concentration of 50 μM. An equal volume of dimethyl sulfoxide (DMSO) was used as the control. In in vivo experiments using mice, YC-1 was administered daily via intraperitoneal injection at a dose of 10 mg/kg/day after dilution in 7.5% bovine serum albumin (BSA). The control group received an equivalent volume of 7.5% BSA via intraperitoneal injection.

### 4.4. Cell Proliferation Assay

Cell proliferation was evaluated using a trypan blue dye exclusion assay. The cells were exposed to PA and/or BOH at directed concentrations and incubated for 48 h under either normoxic or hypoxic conditions. Subsequently, the cells were harvested by trypsinization, and the number was quantified using a TC20 cell counter (Bio-Rad, Hercules, CA, USA). All experiments were conducted in triplicate.

### 4.5. ROS Detection

ROS levels were quantified using a Total ROS Detection Kit (Enzo Life Sciences, New York, NY, USA). Briefly, cells treated with PA and/or BOH were incubated for 48 h under normoxic or hypoxic conditions. Following incubation, cells were stained with an ROS detection reagent per the manufacturer’s protocol. Fluorescence signals were acquired using a FACS Verse flow cytometer (BD Biosciences, Franklin Lakes, NJ, USA) and analyzed using FlowJo software (version 10.7.1, BD Biosciences). Dead cells were excluded by staining with 7-Aminoactinomycin D (BD Biosciences).

### 4.6. ROS Scavenge by N-Acetylcysteine (NAC)

To investigate the involvement of ROS in cell death, N-acetylcysteine (NAC), included in the ROS detection kit (BioVision, Milpitas, CA, USA), was used as an ROS scavenger. NAC was added to the culture medium at the recommended concentration and incubated with the cells under hypoxic conditions for 48 h prior to the analysis.

### 4.7. Flow Cytometric Analysis of Apoptosis

Apoptotic cell death was evaluated using an Annexin V- fluorescein isothiocyanate (FITC) Apoptosis Detection Kit (BioVision, Milpitas, CA, USA). After changing the culture conditions, the cells were incubated under hypoxic conditions for 48 h and subsequently analyzed. After washing with binding buffer, harvested cells were incubated with Annexin V-FITC and propidium iodide (PI) for 5 min. Apoptotic populations were analyzed by flow cytometry based on Annexin V and/or PI positivity. The proportion of apoptotic cells was calculated as the combined percentage of Annexin V–positive/PI–negative and Annexin V–positive/PI–positive populations.

### 4.8. Real-Time Quantitative Reverse Transcription Polymerase Chain Reaction (qRT-PCR)

Total RNA was extracted from cells after incubation at 37 °C for 24 h under either normoxic or hypoxic conditions using an Isogen RNA extraction kit (Nippon Gene, Osaka, Japan). The RNA was subsequently reverse-transcribed (RT) into complementary DNA (cDNA), followed by quantitative polymerase chain reaction (qRT-PCR) according to a previously established protocol [46]. The mRNA expression levels were normalized to β-actin as a reference gene. All experiments were conducted in triplicate. The primer sequences used for qRT-PCR are listed in Table 2.

### 4.9. Western Blotting

Whole-cell extracts from cultured cells and xenograft tumors in mice were prepared using lysis buffer supplemented with a protease inhibitor cocktail, and Western blotting was conducted following the methodology outlined in a previously published protocol 47. The primary antibodies used were HIF-1α (1:1000; cat. no. 610959; BD Biosciences, CA, USA), HEL (Hexanoyl Lysine; 1:1000; cat. no. MHL-021P; JaICA, Shizuoka, Japan), cleaved caspase-3 (1:1000; cat. no. 9661; Cell Signaling Technology, Danvers, MA, USA), ACSL1 (acyl-CoA synthetase long-chain family member 1; 1:1000; cat. no. 13989-1-AP; Proteintech, Rosemont, IL, USA), CPT1A (carnitine palmitoyltransferase 1A; 1:1000; cat. no. 12252; Cell Signaling Technology), MCAD (medium-chain acyl-CoA dehydrogenase; 1:1000; cat. no. 55210-1-AP; Proteintech), LCAD (long-chain acyl-CoA dehydrogenase; 1:2000; cat. no. 17442-1-AP; Proteintech), HADHA (hydroxyacyl-CoA dehydrogenase trifunctional multienzyme complex subunit alpha; 1:50,000; cat. no. 10758-1-AP; Proteintech), HADHB (hydroxyacyl-CoA dehydrogenase trifunctional multienzyme complex subunit beta; 1:12,000; cat. no. 29091-1-AP; Proteintech), ECHS1 (enoyl-CoA hydratase, short chain 1; 1:20,000; cat. no. 29091-1-AP; Proteintech), BDH1 (3-hydroxybutyrate dehydrogenase type 1; 1:10,000; cat. no. 67448-1-lg; Proteintech), HMGCS2 (3-hydroxy-3-methylglutaryl-CoA synthase 2; 1:2000; cat. no. 20940; Cell Signaling Technology), HMGCL (3-hydroxy-3-methylglutaryl-CoA lyase; 1:2000; cat. no. ab197022; abcam, Cambridge, UK), MCT4 (monocarboxylate transporter 4; 1:100,000; cat. no. 22787-1-AP; Proteintech), MCT1 (monocarboxylate transporter 1; 1:3000; cat. no. 85680; Cell Signaling Technology), OXCT1 (3-oxoacid CoA-transferase 1; 1:30,000; cat. no. 67836-1-lg; Proteintech), ACAT1 (acetyl-CoA acetyltransferase 1; 1:10,000; cat. no. 44276; Cell Signaling Technology); ACTB (β-actin; 1:10,000, cat. no. AC15; Sigma-Aldrich; Merck KGaA, MO, USA) was used as the internal control.

### 4.10. In Vivo Nude Mouse Study

Female BALB/c nude mice (age, four weeks) were purchased from Nihon Crea Co. (Tokyo, Japan) and maintained under specific pathogen-free conditions with access to sterile food and water. Body weight and tumor volume were monitored twice per week. Subcutaneous xenograft tumors were established by injecting 3 × 106 Hep3B-SC, Hep3B-HIF1KD, or wild-type Hep3B cells into the flanks of mice. The tumor volume (T) was calculated using the formula T = π/4 × a × b, where a and b represent the shorter and longer tumor axes, respectively, in millimeters. The animals were sacrificed 17 days after changing their diet and drug administration. Under inhalation anesthesia with sevoflurane, mice were euthanized by cardiac puncture, and blood samples were collected. Mice were euthanized immediately under the following conditions: (1) tumor diameter exceeded 20 mm, (2) combined diameters of the right and left tumors exceeded 30 mm, or (3) body weight loss exceeded 20%. CLEA Rodent Diet CE-2 (CLEA Japan, Inc., Tokyo, Japan) was used as a standard diet. According to the manufacturer’s specifications, CE-2 provides approximately 3.48 kcal/g of gross energy. Its composition per kilogram includes approximately 43.6 g of fat, 197.8 g of protein, 443.3 g of carbohydrates, and 47.5 g of fiber.

### 4.11. Ketogenic Diet Feeding

KetoCal^®^ (Nutricia North America, Rockville, MD, USA) was used as a ketogenic diet. KetoCal^®^ is a nutritionally complete ketogenic formula that, according to the manufacturer’s specifications, provides 7.2 Kcal/g of gross energy. Its composition per kilogram includes 720 g of fat, 30 g of carbohydrates, 150 g of protein, and 0 g of fiber. This diet had a ketogenic ratio (fat: protein + carbohydrates) of 4:1, with fat derived from soybean oil. The KetoCal^®^ diet was prepared in paste form by mixing water and KetoCal^®^ at a ratio of 1:2, and was provided to the mice within their cages. In the in vivo experiments, KetoCal^®^ was administered starting 10 days after subcutaneous tumor injection. The diet was replaced every 12 h.

### 4.12. Measurement of Acetyl-CoA in Tumors

After sacrifice, tumors were excised from mice, rapidly frozen in liquid nitrogen, and stored at −80 °C until use. The intratumoral acetyl-CoA concentration was measured using the CheKine Micro Acetyl-CoA Assay Kit (Abbkine, Wuhan, China). In brief, tumors were weighed, and 1 mL of extraction buffer was added per 0.1 g of the tumor tissue. The samples were homogenized on ice, followed by centrifugation at 13,000× *g* for 10 min at 4 °C. The supernatant was collected and analyzed. The concentration of nicotinamide adenine dinucleotide (NADH) was measured as an indirect indicator of acetyl-CoA concentration. This method utilizes a coupling reaction involving malate dehydrogenase and citrate synthase, where acetyl-CoA participates in the reaction to generate NADH. NADH production was quantified by measuring optical density (OD) at 340 nm using a microplate reader. The absorbance value of NADH was represented as the acetyl-CoA concentration in the samples. All measurements were performed in triplicate.

### 4.13. Measurement of Plasma FFA and BOH Levels

The mice were euthanized and blood samples were obtained via cardiac puncture. Each blood sample was centrifuged at 3000× *g* for 10 min at 4 °C, and the plasma supernatant was collected and stored at −80 °C until further use. FFA levels in plasma samples were determined using a Free Fatty Acid Assay Kit (BioAssay Systems, Hayward, CA, USA). A measurement of 10 μL of plasma was mixed with 90 μL of reaction mix. Absorbance at 570 nm was recorded using a microplate reader to determine the OD of the mixture. BOH levels in plasma samples were determined using a Ketone Body Assay Kit (BioAssay Systems). Five microliters of plasma were mixed with 195 μL of the working reagent. The OD of the mixture was measured at 340 nm using a microplate reader. All measurements were performed in triplicate.

### 4.14. Statistical Analysis

All data are presented as the mean ± standard error of the mean (SEM). For comparisons involving more than two groups, one-way analysis of variance (ANOVA) followed by Tukey’s post hoc test was used to evaluate statistical differences. In cases where only two groups were compared (e.g., control vs. KD + HIF-1α inhibition), an unpaired Student’s *t*-test was applied. *p*-values of less than 0.05 were considered to indicate statistical significance. All statistical analyses were conducted using JMP Pro (version 16, SAS Institute, Inc., Cary, NC, USA).

### 4.15. Approval for Animal Experiments

All animal experiments were approved by the Animal Experimentation Ethics Committee of Saga University (Saga, Japan; Approval No. A2021-036-0) and were conducted in accordance with the guidelines and regulations of the committee. This study is reported in accordance with the ARRIVE guidelines.

## Figures and Tables

**Table 1:** The expression ratio of mRNA and quantification ratio of protein expression in Hep3B, and regulation by HIF1 under hypoxic conditions.

Gene	Function in Metabolism	Expression Ratio of mRNA		Quantification Ratio of Protein Expression		Regulation by HIF-1 Under Hypoxia
		H/N Ratio in SC	KD/SC Ratio Under Hypoxia	H/N Ratio in SC	KD/SC Ratio Under Hypoxia	
*ACSL1*	FAO	0.773 ± 0.016	1.407 ± 0.040	0.629	1.192	downregulation
*CPT1*		0.443 ± 0.044	1.396 ± 0.129	0.640	1.937	
*MCAD*		0.042 ± 0.001	12.18 ± 0.483	0.640	1.127	
*LCAD*		0.563 ± 0.027	0.976 ± 0.047	0.586	1.081	
*HADHA*		0.376 ± 0.038	1.77 ± 0.234	0.475	1.526	
*HADHB*		0.354 ± 0.051	1.940 ± 0.199	0.619	1.336	
*ECHS1*		0.551 ± 0.032	2.662 ± 0.228	0.522	1.276	
*HMGCS2*	Ketogenesis	3.566 ± 0.466	0.607 ± 0.170	1.724	0.575	upregulation
*HMGCL*		4.761 ± 0.444	0.588 ± 0.010	2.075	0.426	
*MCT4*		4.324 ± 0.713	0.436 ± 0.044	1.871	0.564	
*MCT1*	Ketolysis	0.610 ± 0.056	1.483 ± 0.081	0.782	2.163	downregulation
*OXCT1*		0.417 ± 0.038	2.556 ± 0.404	0.655	1.861	
*ACAT1*		0.364 ± 0.011	4.219 ± 0.694	0.616	2.030	

**Table 2:** Primer sequences for multiple genes.

Genes	Forward Primer	Reverse Primer
*ACSL1*	5′-CGA CGA GCC CTT GGT GTA TTT-3′	3′-GGT TTC CGA GAG CCT AAA CAA-5′
*CPT1*	5′-ATC AAT CGG ACT CTG GAA ACG G-3′	3′-TCA GGG AGT AGC GCA TGG T-5′
*MCAD*	5′-TGG GAG GTT GAT TCT GGT GGT CG-3′	3′-TGT CAA TGT GTT CAC GGG CT-5′
*LCAD*	5′-TTG GCA AAA CAG TTG CTC AC-3′	3′-ACA TGT ATC CCC AAC CTC CA-5′
*HADHA*	5′-TTG AAA AGG CCG ACA TGG TG-3′	3′-AGT GAT CTG GAA TCA CCG CTT-5′
*HADHB*	5′-ACA CTG GTT TCT GGT TGG CTC-3′	3′-GAT GCA ACA AAC CCG TAA GCG-5′
*ECHS1*	5′-GCT GCT GTC AAT GGC TAT GC-3′	3′-TCT TTC CGG TCA TCA GTG GC-5′
*HMGCS2*	5′-ATC AAC TCC CTG TGC CTG AC-3′	3′-GTA CCA CCG TAG CAG GCA TT-5′
*HMGCL*	5′-GCA CCT CAT CTA TGG GCA CT-3′	3′-GGG TAG TTG ATG CCA GGA AA-5′
*MCT4*	5′-CCA TGC TCT ACG GGA CAG G-3′	3′-GCT TGC TGA AGT AGC GGT T-5′
*MCT1*	5′-AGT AGT TAT GGG AAG AGT CAG CA-3′	3′-GTC GGG CTA CCA TGT CAA CA-5′
*OXCT1*	5′-GGG TCC ATA TCC ACG ACA AC-3′	3′-CCA GTT AGC CAG GTC ACC AT-5′
*ACAT1*	5′-GGC TGG TGC AGG AAA TAA GA-3′	3′-GGA ATC CCT GCC TTT TCA AT-5′
*ACTB*	5′-ACG CCT CTG GCC GTA CCA CT-3′	3′-TAA TGT CAC GCA CGA TTT CCC-5′
*BDH1*	5′-ACC TGC CAG CTA AGA ACC AC-3′	3′-ATC TCA TAG CGC AGG CAG TC-5′

## Data Availability

The original contributions presented in this study are included in the article/supplementary material. Further inquiries can be directed to the corresponding author.

## Supplementary Materials

The following supporting information can be downloaded at https://www.mdpi.com/article/10.3390/ijms262411769/s1.

## Abbreviations

The following abbreviations are used in this manuscript:

HIF-1	Hypoxia-inducible factor-1
KGD	Ketogenic diet
HCC	Hepatocellular carcinoma
KD	Knockdown
SC	Scramble control
PA	Palmitic acid
ROS	Reactive oxygen species
BOH	β-hydroxybutyrate
FAO	Fatty acid oxidation
FFA	Free fatty acid
AcAc	Acetoacetate
OXPHOS	Oxidative phosphorylation
ETC	Electron transport chain
NAC	N-acetylcysteine
ACSL1	Acyl-CoA synthetase long-chain family member 1
CPT1A	Carnitine palmitoyltransferase 1A
MCAD	Medium-chain acyl-CoA dehydrogenase
LCAD	Long-chain acyl-CoA dehydrogenase
HADHA	Hydroxyacyl-CoA dehydrogenase trifunctional multienzyme complex subunit alpha
ECHS1	Enoyl-CoA hydratase, short chain 1
HADHB	Hydroxyacyl-CoA dehydrogenase trifunctional multienzyme complex subunit beta
HMGCS2	3-hydroxy-3-methylglutaryl-CoA synthase 2
HMGCL	3-hydroxy-3-methylglutaryl-CoA lyase
BDH1	β-hydroxybutyrate dehydrogenase type 1
MCT4	Monocarboxylate transporter 4
MCT1	Monocarboxylate transporter 1
OXCT1	3-oxoacid CoA-transferase 1
ACAT1	Acetyl-CoA acetyltransferase
HEL	Hexanoyl lysine
TG	Triglyceride
YC-1	3-(5′-hydroxymethyl-2′-furyl)-1-benzylindazole
HRE	Hypoxic response element
BDH	β-hydroxybutyrate dehydrogenase
DMEM	Dulbecco’s Modified Eagle Medium
FBS	Fetal bovine serum
shRNA	Short hairpin RNA
BSA	Bovine serum albumin
DMSO	Dimethyl sulfoxide
FITC	Fluorescein isothiocyanate
PI	Propidium iodide
RT	Reverse-transcribed
cDNA	Complementary DNA
qRT-PCR	Quantitative polymerase chain reaction
OD	Optical density
NADH	Nicotinamide adenine dinucleotide
SEM	Standard error of the mean
ANOVA	Analysis of variance
ACTB	β-actin
