# Supplementary material for: Putative Role of Fatty Acid Metabolic Therapy Using Ketogenic Diet and HIF-1α Inhibition in Hepatocellular Carcinoma: Evidence from an In Vitro Study
Source: Int J Mol Sci. 2025 Dec 5;26(24):11769. doi: 10.3390/ijms262411769 (PMC12732810; doi:10.3390/ijms262411769)
Supplement: Supplementary file 1 [file ijms-26-11769-s001.zip › ijms-3930255-supplementary.pdf]

Supplementary Material

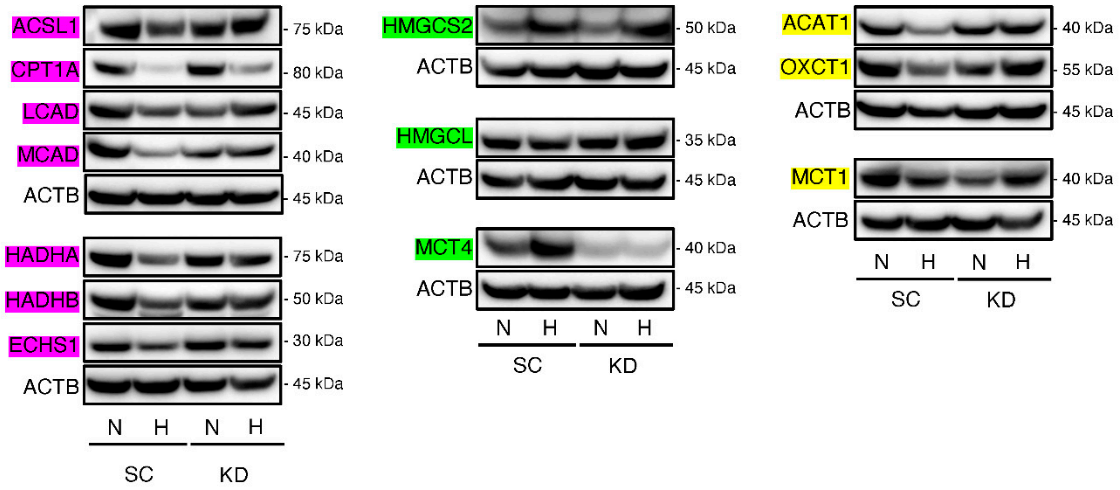

Figure S1. The analysis of the expression of key enzymes related to fatty acid oxidation (FAO) and ketone body metabolism in SC and HepG2-HIF-1αKD cells under normoxic and hypoxic conditions. Western blotting of key enzymes in SC and KD cells under normoxic or hypoxic conditions for 48 hours.

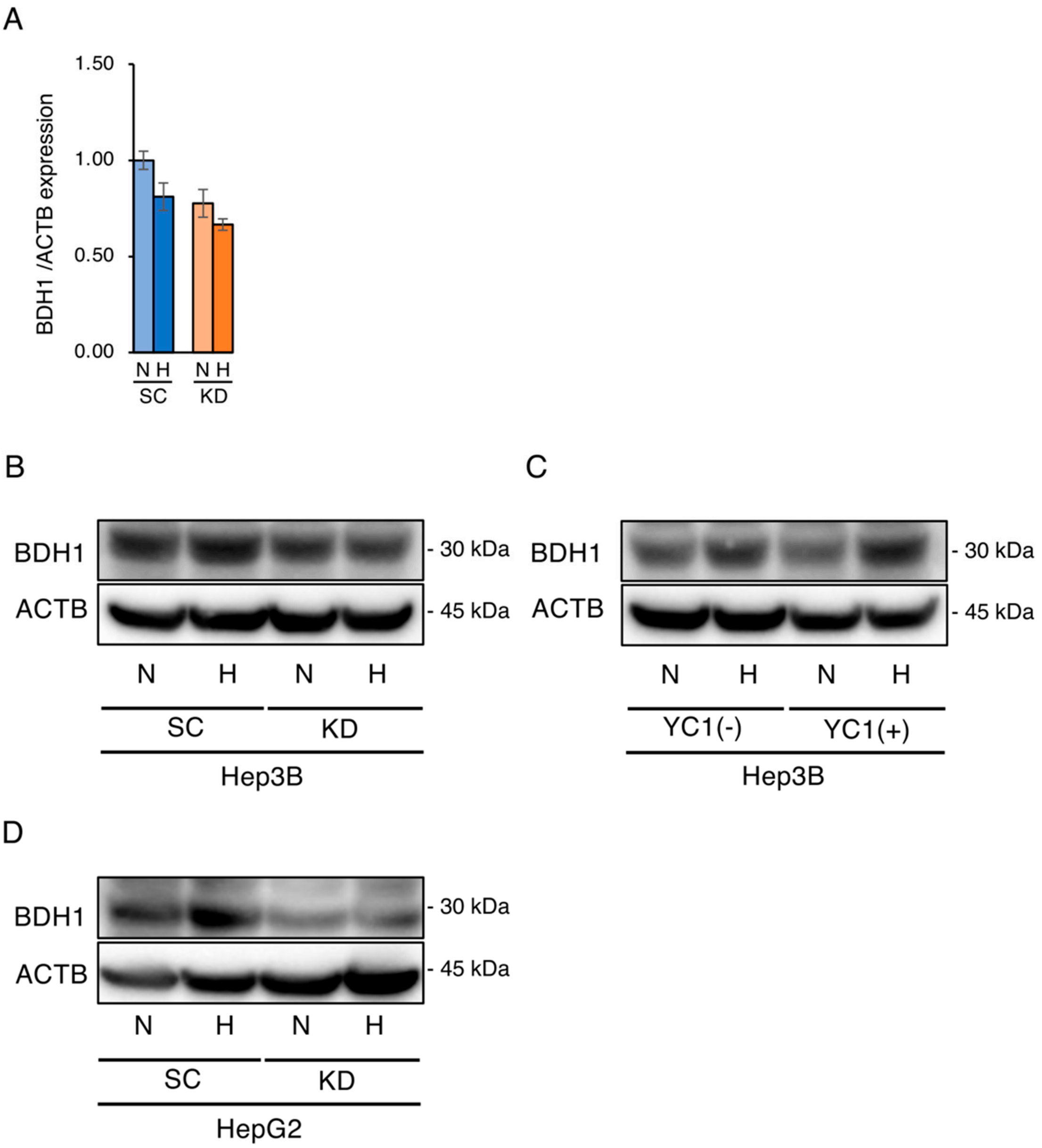

Figure S2. Analysis of the expression of BDH1 in SC and KD cells under normoxic and hypoxic conditions in Hep3B. A. qRT-PCR of BDH1 in SC and KD cells maintained under normoxic (N) or hypoxic (H) conditions for 24 h. B. Western blotting of BDH1 in SC and Hep3B HIF-1αKD cells maintained under normoxic or hypoxic conditions for 48 h. C. Western blotting to analyze the HIF-1α protein expression in wild-type Hep3B cells with or without YC-1 (50 μM) under normoxic (N) or hypoxic (H) conditions for 48 h. D. Western blotting of BDH1 in SC and HepG2 HIF-1αKD cells maintained under normoxic or hypoxic conditions for 48 h.

The quantification ratio of protein expression in HepG2, and regulation by HIF1 under hypoxic conditions.

Gene	Function in metabolism	Quantification ratio of protein expression		Regulation by HIF-1 under hypoxia
		H/N ratio in SC	KD/SC ratio under hypoxia	
ACSL1	FAO	0.682	1.522	downregulation
CPT1		0.287	2.140	
MCAD		0.241	2.896	
LCAD		0.532	1.688	
HADHA		0.401	2.122	
HADHB		0.646	1.660	
ECHS1		0.674	1.893	
HMGCS2	Ketogenesis	0.826	1.128	variable
HMGCL		0.773	1.630	
MCT4		1.964	0.178	
MCT1	Ketolysis	0.726	1.533	downregulation
OXCT1		0.897	1.007	
ACAT1		0.662	1.443	

Table S1. The quantification ratio of protein expression, which estimated using KD and SC cells, and regulation by HIF1 in HepG2 under hypoxic conditions.
